# Supplementary material for: Heterogeneously deacetylated chitosans possess an unexpected regular pattern favoring acetylation at every third position
Source: Nat Commun. 2024 Aug 6;15:6695. doi: 10.1038/s41467-024-50857-1 (PMC11303684; doi:10.1038/s41467-024-50857-1)
Supplement: Supplementary file 1 — Supplementary Information [file 41467_2024_50857_MOESM1_ESM.pdf]

Supplementary information

Heterogeneously deacetylated chitosans possess an unexpected regular pattern favoring acetylation at every third position

Margareta J. Hellmann[1], Dominique Gillet[2], Stéphane Trombotto[3], Sonja Raetz[1], Bruno M. Moerschbacher[1*], Stefan Cord-Landwehr[1]

[1]Institute for Biology and Biotechnology of Plants, University of Münster, 48143 Münster, Germany
[2]Gillet Chitosan SAS, La Ville Es Comte, 22350 Plumaudan, France
[3]Ingénierie des Matériaux Polymères (IMP), UMR 5223, Université Claude Bernard Lyon 1, CNRS, INSA Lyon, Université Jean Monnet Saint-Etienne, F-69622 Villeurbanne, France

*moersch@uni-muenster.de

Chitosan materials

Supplementary Table 1: Details of the production method, fraction of acetylation (FA) and molecular weight (MW) of chitosan polymers used in this study. For HTDA chitosans, the type of chitin starting material (α or β) is indicated along with the source organism. The FAs were determined by enzymatic MS fingerprinting developed for soluble samples[1] and (in parentheses) for insoluble samples[2], and the weight and number average molecular weights (M_w and M_n, respectively) and MW dispersity ($Đ_M$) were determined by SEC-MALLS-RI[3]. Deacetylated samples were kindly provided by (a) Dr. Katja Richter (Heppe Medical Chitosan, Germany), (b) Dr. Mats Andersson (Flexichem AB, Sweden), (c) the former group of Prof. Kjell Vårum (Norwegian University of Science and Technology, Trondheim), and (d) Gillet Chitosan SAS (France), or (e) prepared in-house. Samples labeled *series* were collected during HTDA. Series 1 and 2A were not subject to further chemical treatment, whereas series 2B samples are series 2A after chemical depolymerization. *N*-acetylated samples were produced in-house in Münster from fully deacetylated 134 chitosan (f), in-house in Lyon (g), or provided again by HMC (a). Commercially available samples are marked with an asterisk (*). HTDA, heterogeneously deacetylated; HMDA, homogeneously deacetylated; CNA, chemically *N*-acetylated; CO, *Chionoecetes opilio*; PB, *Pandalus borealis*; P, *Penaeus* spp.; LD, *Loligo duvauceli*.

Sample	Method	Chitin	FA	M_w [kDa]	M_n [kDa]	$Đ_M$
111[d*]	HTDA	Squid, LD (β)	0.224	655.0		1.40
112[d*]	HTDA	Squid, LD (β)	0.108	448.7		1.35
114[d*]	HTDA	Squid, LD (β)	0.061	496.3		1.47
134[d*]	HTDA	Squid, LD (β)	0.003	244.0		1.58
134_0.06[f]	CNA	-	0.071	243.9		2.03
134_0.1[f]	CNA	-	0.11			
134_0.10[f]	CNA	-	0.113	210.8		2.92
134_0.11[f]	CNA	-	0.110	343.4		4.48
134_0.15_K[f]	CNA	-	0.15			
134_0.19[f]	CNA	-	0.176	114.4		
134_0.2[f]	CNA	-	0.18			
134_0.20[f]	CNA	-	0.214			
134_0.20g[f]	CNA	-	0.180	196.9		3.27
134_0.27[f]	CNA	-	0.315	176.4		1.85
134_0.29[f]	CNA	-	0.316	34.2	23.4	1.52
134_0.3[f]	CNA	-	0.32			
134_0.32_K[f]	CNA		0.32			
134_0.32[f]	CNA	-	0.359	205.5		2.34

**Sample**	**Method**	**Chitin**	**FA**	M_w **[kDa]**	M_n **[kDa]**	$Đ_M$
134_0.35g[f]	CNA	-	0.374			
134_0.4[f]	CNA	-	0.43			
134_0.42[f]	CNA	-	0.424	251.3		1.86
134_0.50[f]	CNA	-	0.428	203.5		1.83
651[d]	HTDA	Shrimp, P (α)	0.219	133.9	85.3	1.57
652[d]	HTDA	Shrimp, P (α)	0.084	77.2		2.19
70/100[a]*	HTDA	Snow crab, CO (α)	0.248	196.0	94.3	2.09
70/1000[a]*	HTDA	Snow crab, CO (α)	0.256	351.8	198.7	1.94
70/20[a]*	HTDA	Snow crab, CO (α)	0.233	95.3	57.4	1.74
75/20[a]*	HTDA	Snow crab, CO (α)	0.239	113.1	58.5	2.02
80/20[a]*	HTDA	Snow crab, CO (α)	0.173	106.3	79.2	1.36
80/5[a]*	HTDA	Snow crab, CO (α)	0.112	19.3	8.5	2.85
85/20[a]*	HTDA	Snow crab, CO (α)	0.088	78.0	58.3	1.39
90/20[a]*	HTDA	Snow crab, CO (α)	0.079	77.0	53.5	1.44
95/20[a]*	HTDA	Snow crab, CO (α)	0.032	88.5	32.1	2.83
FPT_0.2[e]	FPT HTDA	Squid, LD (β)	0.180			
FPT_0.585[e]	FPT HTDA	Squid, LD (β)	0.470			
HMC_DDA33[a]	CNA	-	0.571	217.8	156.7	1.44
HMC_DDA50[a]	CNA	-	0.421	132.6	78.9	1.74
HMC_DDA68[a]	CNA	-	0.243	169.1	73.0	2.36
HMC_DDA80[a]	CNA	-	0.145	129.8	48.5	2.69
HMC_DDA83[a]	CNA	-	0.117	178.7	70.7	2.53
L10[g]	CNA	-	0.374	170.4	80.9	2.21
L11[g]	CNA	-	0.356	530.8	351.3	1.51
L8[g]	CNA	-	0.500	215.9	94.9	2.19
L9[g]	CNA	-	0.445	621.4	358.0	2.15
shrimp_chitin[e]	-	Shrimp, P (α)	(0.95)	-	-	-
shrimp_020min[e]	HTDA	Shrimp, P (α)	0.445	369.7	171.3	2.18
shrimp_040min[e]	HTDA	Shrimp, P (α)	0.368	294.0	111.3	2.66
shrimp_060min[e]	HTDA	Shrimp, P (α)	0.344	294.3	107.6	2.78
shrimp_080min[e]	HTDA	Shrimp, P (α)	0.325	347.0	154.4	2.46
shrimp_100min[e]	HTDA	Shrimp, P (α)	0.316	265.9	102.6	2.60
shrimp_120min[e]	HTDA	Shrimp, P (α)	0.300	252.1	124.6	2.05
shrimp_140min[e]	HTDA	Shrimp, P (α)	0.297	235.8	96.3	2.51
squid_chitin[e]	-	Squid, LD (β)	(0.93)	-	-	-
squid_1_020min[e]	HTDA	Squid, LD (β)	0.242			
squid_1_040min[e]	HTDA	Squid, LD (β)	0.189			
squid_1_061min[e]	HTDA	Squid, LD (β)	0.160			
squid_1_081min[e]	HTDA	Squid, LD (β)	0.149			
squid_1_090min[e]	HTDA	Squid, LD (β)	0.157			
squid_2A_020min[e]	HTDA	Squid, LD (β)	0.284	499.5	434.9	1.14
squid_2A_040min[e]	HTDA	Squid, LD (β)	0.253	245.1	199.5	1.30

Sample	Method	Chitin	FA	M_w [kDa]	M_n [kDa]	$Đ_M$
squid_2A_060min[e]	HTDA	Squid, LD (β)	0.211	535.5	303.3	1.81
squid_2A_080min[e]	HTDA	Squid, LD (β)	0.196	550.1	324.8	1.77
squid_2A_090min[e]	HTDA	Squid, LD (β)	0.174	685.6	457.8	1.50
squid_2A_110min[e]	HTDA	Squid, LD (β)	0.061	705.1	523.4	1.35
squid_2A_130min[e]	HTDA	Squid, LD (β)	0.054	788.4	568.2	1.40
squid_2A_135min[e]	HTDA	Squid, LD (β)	0.044	683.9	438.3	1.56
squid_2B_020min[e]	HTDA	Squid, LD (β)	0.275	27.5	20.6	1.32
squid_2B_040min[e]	HTDA	Squid, LD (β)	0.230	50.1	36.7	1.35
squid_2B_060min[e]	HTDA	Squid, LD (β)	0.201	58.7	41.2	1.44
squid_2B_080min[e]	HTDA	Squid, LD (β)	0.169	125.5	70.5	1.80
squid_2B_090min[e]	HTDA	Squid, LD (β)	0.158	203.4	114.5	1.78
squid_2B_110min[e]	HTDA	Squid, LD (β)	0.060	122.6	81.5	1.51
squid_2B_130min[e]	HTDA	Squid, LD (β)	0.048	140.6	84.5	1.67
squid_2B_135min[e]	HTDA	Squid, LD (β)	0.046	130.5	75.5	1.73
T_DA16[c]	HMDA[4,5]	Shrimp, PB (α)	0.148	34.6		3.97
T_DA34[c]	HMDA[4,5]	Shrimp, PB (α)	0.334	43.2		1.03
Viscosan_DDA40[b]*	HMDA		0.551	341.7		1.61
Viscosan_DDA46[b]*	HMDA		0.509	171.9		1.35
Viscosan_DDA48[b]*	HMDA		0.479	271.6		1.74
Viscosan_DDA52[b]*	HMDA		0.466	167.8		1.44
Viscosan_DDA59[b]*	HMDA		0.402	393.6		1.41
Viscosan_DDA69[b]*	HMDA		0.318	442.5	227.0	1.97

AaChio products on FA = 0.32: product profiles and block sizes

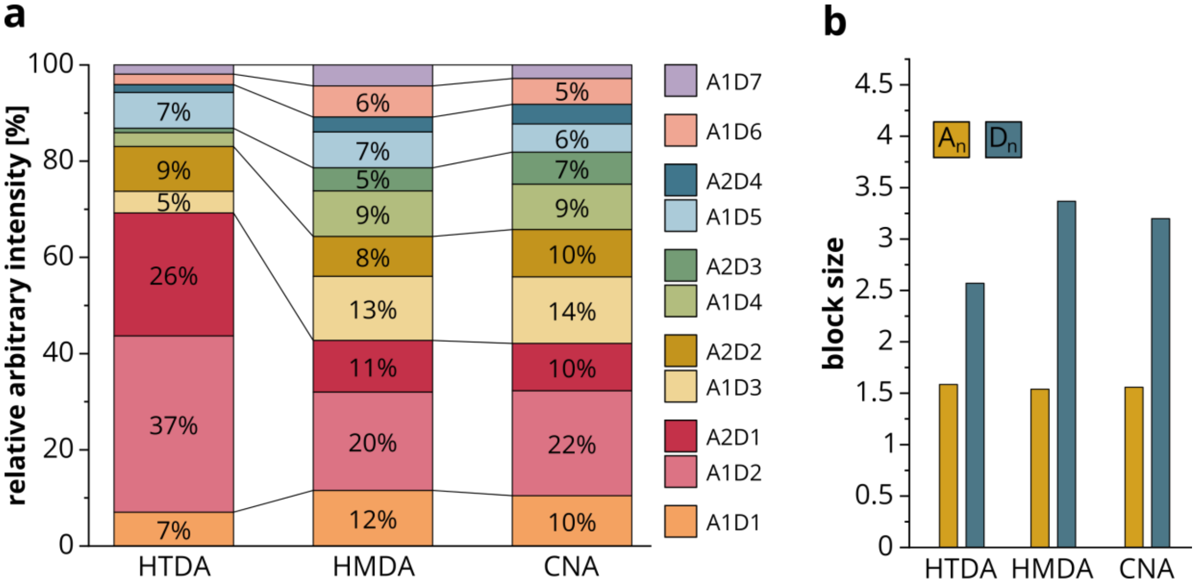

Supplementary Figure 1: Products of the AaChio-catalyzed hydrolysis of HTDA, HMDA and CNA chitosans (FA = 0.32). Details of the chitosan samples are provided in Supplementary Table 1 (HTDA, shrimp_100min; HMDA, Viscosan_DDA69; CNA, 134_0.29). **a.** Profile of the products with a DP of 2–8 based on MS signal intensities. The percentages do not reflect the relative amount of each oligomer due to differences in the ionization efficiency. **b.** Number average A-block and D-block sizes (A_n and D_n, respectively) calculated from the MS signals of the products[6] (see Block sizes section, Supplementary Eq. (1) and Eq. (2), below). HTDA, heterogeneously deacetylated; HMDA, homogeneously deacetylated; CNA, chemically *N*-acetylated.

AaChio products on FA = 0.17: RI chromatograms

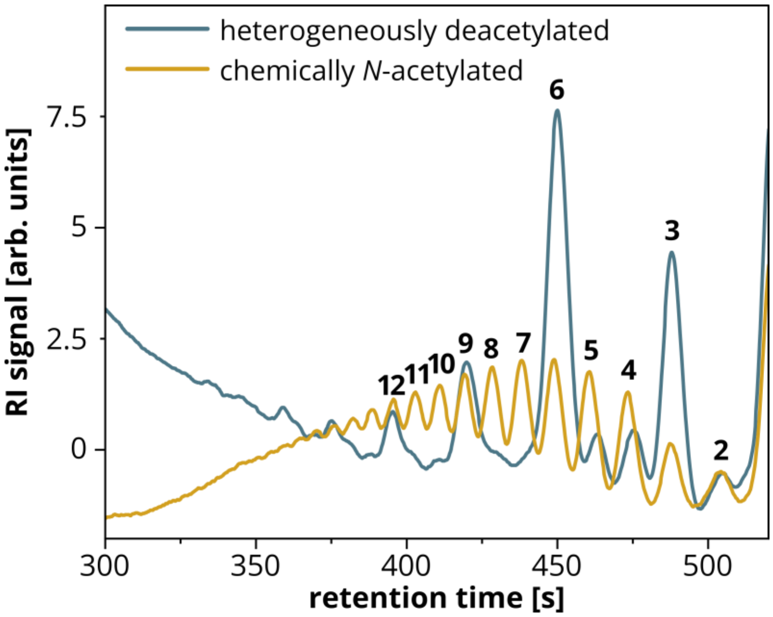

Supplementary Figure 2: Products of the AaChio-catalyzed hydrolysis of HTDA or CNA chitosans (FA = 0.17, M_w of ~110 kDa). Details of the chitosan samples are provided in Supplementary Table 1 (heterogeneously deacetylated, 80/20; chemically *N*-acetylated, 134_0.19). The refractive index (RI) signals of oligomer products with the indicated DP are shown after size exclusion chromatography. HTDA, heterogeneously deacetylated; CNA, chemically *N*-acetylated.

Heterogeneous deacetylation over time

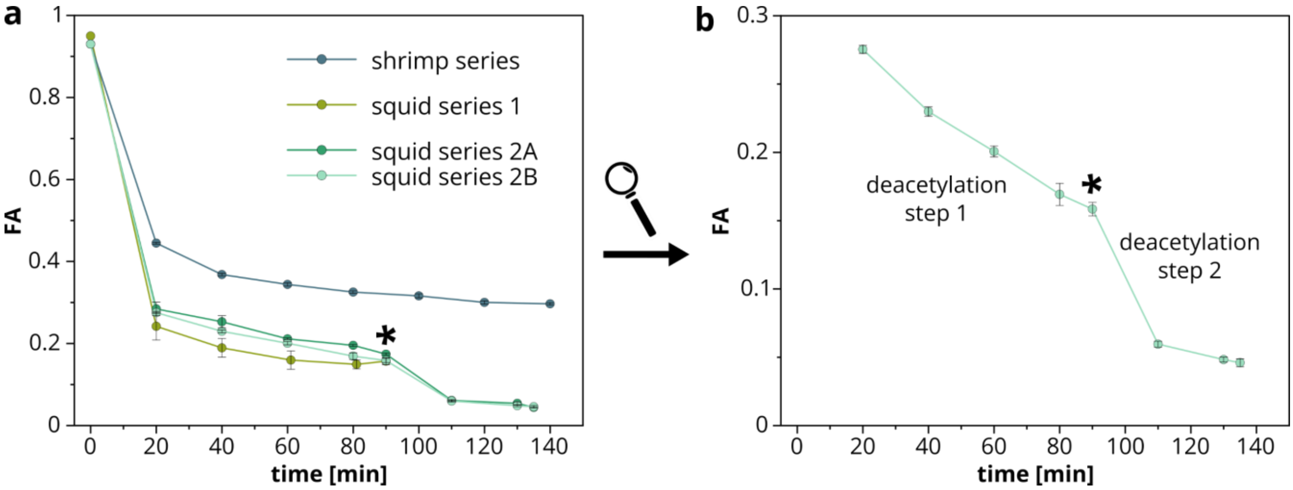

Supplementary Figure 3: Fractions of acetylation (FAs) of consecutive samples taken during the heterogeneous deacetylation of chitin from shrimp or squid. The FAs of the starting materials were determined by enzymatic MS fingerprinting for insoluble samples[2], whereas only the soluble part was analyzed for the other samples[1] (two technical replicates (n = 2) of two biological replicates each (N = 2), SD indicated). The asterisk (*) marks the time point at 90 min, after which the series 2 chitosan was removed from the reactor, rinsed and subsequently, the second deacetylation was started. Squid series 1 and 2A were not subject to further chemical treatment, whereas series 2B samples are series 2A after chemical depolymerization. Details of the chitosan samples are provided in Supplementary Table 1. **a.** Comparison of all four series from chitin to the final chitosan product. **b.** Zoom in on squid series 2B without showing the chitin starting material.

Block sizes

The number average sizes of A-blocks and D-blocks were determined based on enzymatic cleavage products[6], exploiting the exceptionally high subsite specificities of the chitinosanase from *Alternaria alternata* (AaChio)[7]. AaChio is absolutely specific for D-units at subsite -2 and for A-units at subsite -1, but lacks specificity at subsites +1 and +2. This enzyme therefore hydrolyzes chitosans precisely after each A-unit that is preceded by a D-unit, leading to final products with the sequence $A_{x-1}D_{y-1}DA$ (x = total number of A-units, y = total number of D-units) that originate from chitosan polymer cleavage at motif ···DA|$A_{x-1}D_{y-1}$DA|··· (Supplementary Fig. 4). Hence, the number of A- or D-units within an AaChio product directly corresponds to the length of A- or D-blocks at the cleaved site (e.g., A_3D_4 = AADDDDA originating from ···D**A|AADDDD**A|···; A-block size: 3, D-block size: 4).

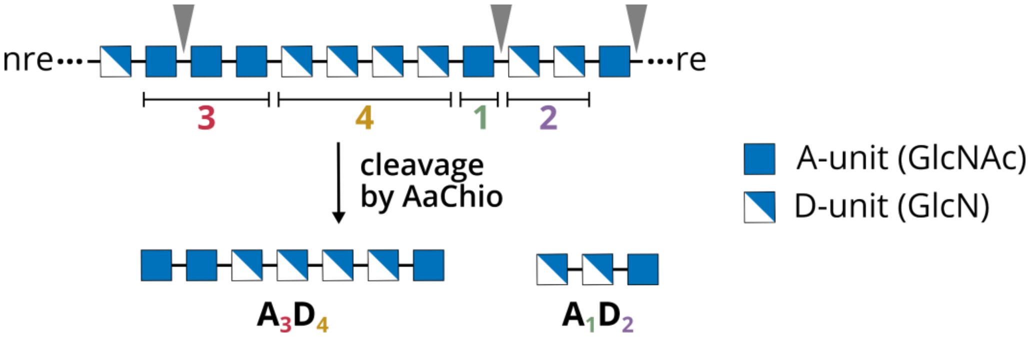

Supplementary Figure 4: Connection between the products of AaChio and the A- and D-block sizes of the cleaved polymer. An exemplary section of a chitosan substrate is shown from non-reducing end (nre) to reducing end (re), the lengths of the A- or D-blocks are indicated. The composition of the final products of AaChio cleavage after the motif DA directly reveals the block sizes within the original polymer substrate.

Based on the quantified oligomer products, there are two ways to calculate average block sizes – weight average or number average. The weight average takes into account the weight fraction of the oligomers corresponding to a certain block size, whereas the number average considers their molar fraction. In simple terms, this means that each molecule is given the same weight in the number average, whereas heavier oligomers are weighted more than lighter ones in the weight average. As a result, the weight average block sizes are always larger than their number average counterparts. There is no clear reason why one should choose the weight average over the number average or vice versa – we chose number average for this study.

(1) $$block(A)_n = \sum_i I_i * N(A)_i$$

(2) $$block(D)_n = \sum_i I_i * N(D)_i$$

$block(A)_n/(D)_n$: number average A-block/D-block size
I: relative oligomer intensity
N(A)/N(D): number of A-units/D-units in oligomer
for each detected oligomer i

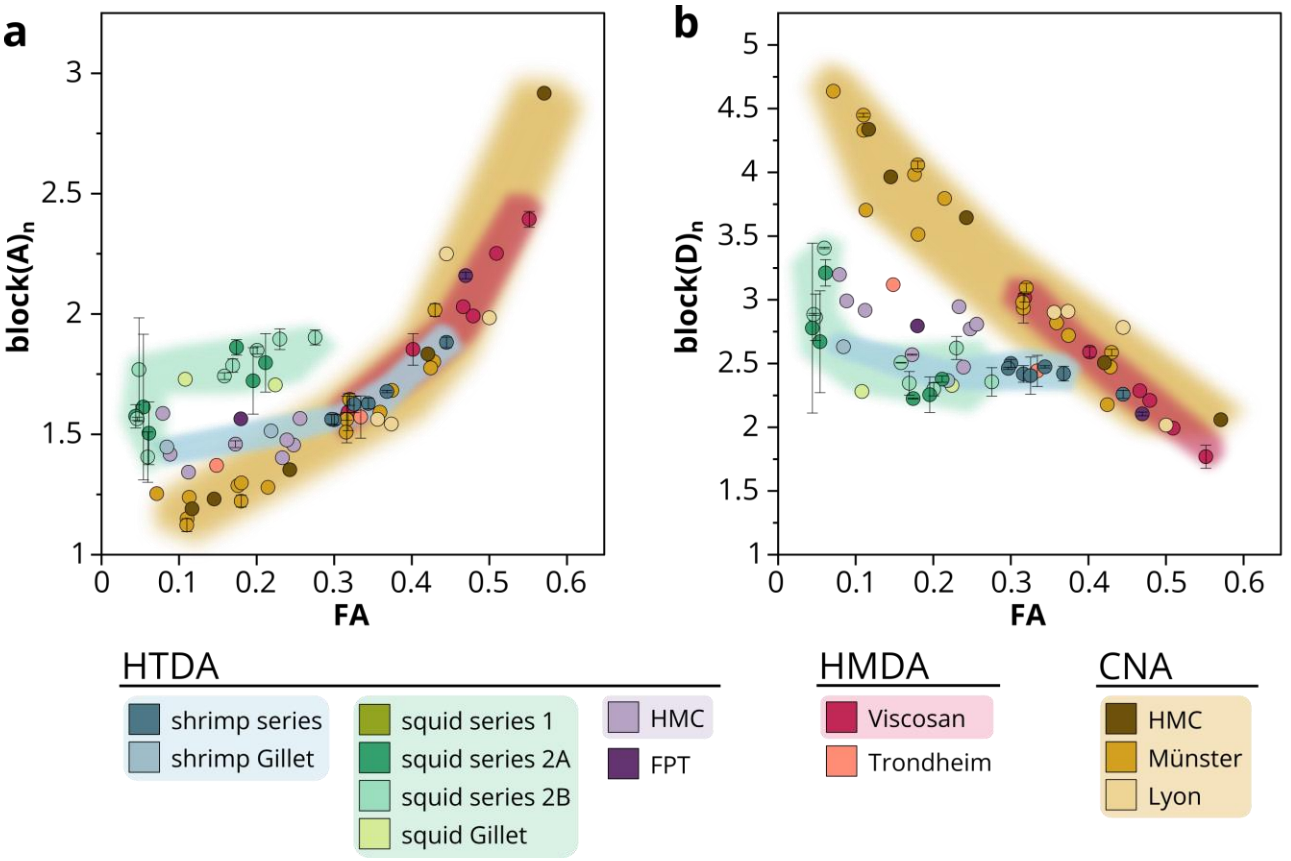

Supplementary Figure 5: Number average block sizes of chitosans produced by different methods following hydrolysis by AaChio. Details of the chitosan samples are provided in Supplementary Table 1. Samples labeled *series* were collected during heterogeneous deacetylation. Series 1 and 2A were not subject to further chemical treatment, whereas series 2B samples are series 2A after chemical depolymerization. Although the determination of triad strength is possible with very small amounts of AaChio products, a reliable calculation of the block sizes requires sufficiently strong MS signals. Therefore, only samples with a total MS signal intensity > 3.5×10^8 were considered. **a.** Number average A-block sizes A_n calculated from the MS signals of the products[6] using Supplementary Equation (1). **b.** Number average D-block sizes D_n calculated from the MS signals of the products[6] using Supplementary Equation (2). For averages of multiple replicates, the standard deviation is indicated, and the number of replicates is accessible in the corresponding source data of this figure at https://doi.org/10.17879/46918475258. HTDA, heterogeneously deacetylated; HMDA, homogeneously deacetylated; CNA, chemically *N*-acetylated; HMC, Heppe Medical Chitosan; FPT, freeze-pump out-thaw.

Hydrolysis of FA = 0.32: Product profiles and efficiency

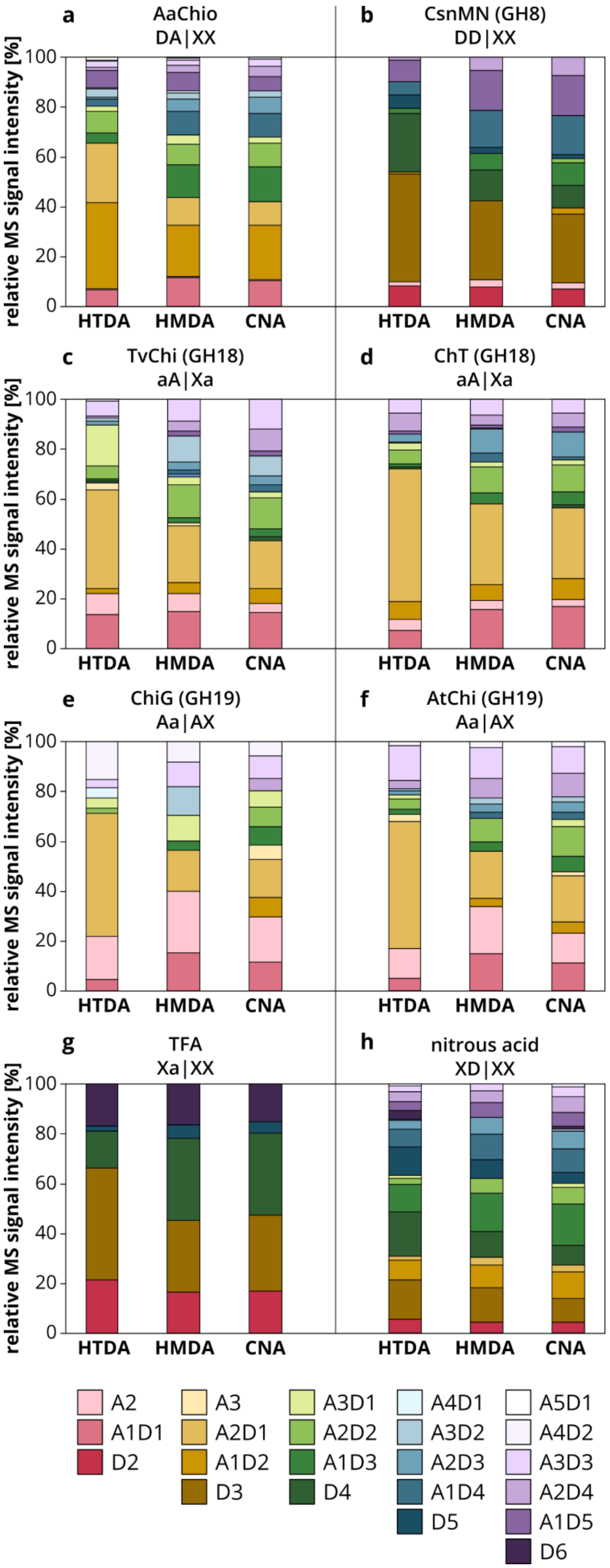

Supplementary Figure 6: Products of HTDA, HMDA and CNA chitosans (FA = 0.32) digested with different enzymes. Details of the chitosan samples are provided in Supplementary Table 1 (HTDA, shrimp_100min; HMDA, Viscosan_DDA69; CNA, 134_0.29). The profiles of products with a DP of 2–6 are shown based on their MS signal intensities. The percentages do not reflect the relative amount of each oligomer due to differences in ionization efficiency. The chitosans were enzymatically hydrolyzed using **a.** chitinosanase from *Alternaria alternata*[7] (AaChio), **b.** chitosanase from *Bacillus* sp. MN[8–10] (CsnMN), **c.** chitinase from *Trichoderma virens*[11] (TvChi), **d.** human chitotriosidase[12–14] (ChT), **e.** chitinase from *Streptomyces coelicolor* A3(2)[15] (ChiG) and **f.** chitinase from *Arabidopsis thaliana* (TAIR: AT3G54420) (AtChi), or partially chemically degraded by **g.** acidic hydrolysis using trifluoroacetic acid (TFA), or **h.** nitrous acid deamination. The subsite preferences of the enzymes from subsite -2 to +2 are indicated by X (no strong preference), A (absolute specificity for A), a (considerable preference for A), D (absolute specificity for D) or d (considerable preference for D). Preferential chemical acidic hydrolysis after A-units[16,17] by TFA (corresponding to a considerable A-preference at subsite -1) as well as the cleavage by nitrous acid deamination[18,19] exclusively after D-units[20] (corresponding to an absolute D-specificity at subsite -1) are indicated accordingly. HTDA, heterogeneously deacetylated; HMDA, homogeneously deacetylated; CNA, chemically *N*-acetylated.

Supplementary Table 2: Efficiency of enzymatic or chemical cleavage of differently produced chitosans of FA 0.32 quantified as a percentage of products with a DP ≤ 12. Quantification is based on the integrated RI signals. The chitosans were enzymatically hydrolyzed using chitinosanase from *Alternaria alternata*[7] (AaChio), chitosanase from *Bacillus* sp. MN[8–10] (CsnMN), chitinase from *Trichoderma virens*[11] (TvChi), human chitotriosidase[12–14] (ChT), chitinase from *Streptomyces coelicolor* A3(2)[15] (ChiG), or chitinase from *Arabidopsis thaliana* (TAIR: AT3G54420) (AtChi). Partial chemical acidic hydrolysis was carried out using trifluoroacetic acid (TFA), partial cleavage by deamination was performed with nitrous acid. The subsite preferences of the enzymes from subsite -2 to +2 are indicated by X (no strong preference), A (absolute specificity for A), a (considerable preference for A), D (absolute specificity for D) or d (considerable preference for D). Preferential chemical acidic hydrolysis after A-units[16,17] by TFA (corresponding to a considerable A-preference at subsite -1) as well as the cleavage by nitrous acid deamination[18,19] exclusively after D-units[20] (corresponding to an absolute D-specificity at subsite -1) are indicated accordingly. HTDA, heterogeneously deacetylated; HMDA, homogeneously deacetylated; CNA, chemically *N*-acetylated.

Cleavage by	Substrate	Products DP ≤ 12 [%]	Products DP > 12 [%]
**AaChio DA|XX**	HTDA	73	27
	HMDA	88	12
	CNA	90	10
**CsnMN DD|XX (GH8)**	HTDA	42	58
	HMDA	46	54
	CNA	39	61
**TvChi aA|Xa (GH18)**	HTDA	28	72
	HMDA	41	59
	CNA	48	52
**ChT aA|Xa (GH18)**	HTDA	42	58
	HMDA	55	45
	CNA	60	40
**ChiG Aa|AX (GH19)**	HTDA	20	80
	HMDA	9	91
	CNA	13	87
**AtChi Aa|AX (GH19)**	HTDA	22	78
	HMDA	19	81
	CNA	24	76
**TFA Xa|XX**	HTDA	52	48
	HMDA	90	10
	CNA	94	6
**nitrous acid XD|XX**	HTDA	53	47
	HMDA	18	82
	CNA	40	60

Hydrolysis of FA = 0.17: RI chromatograms

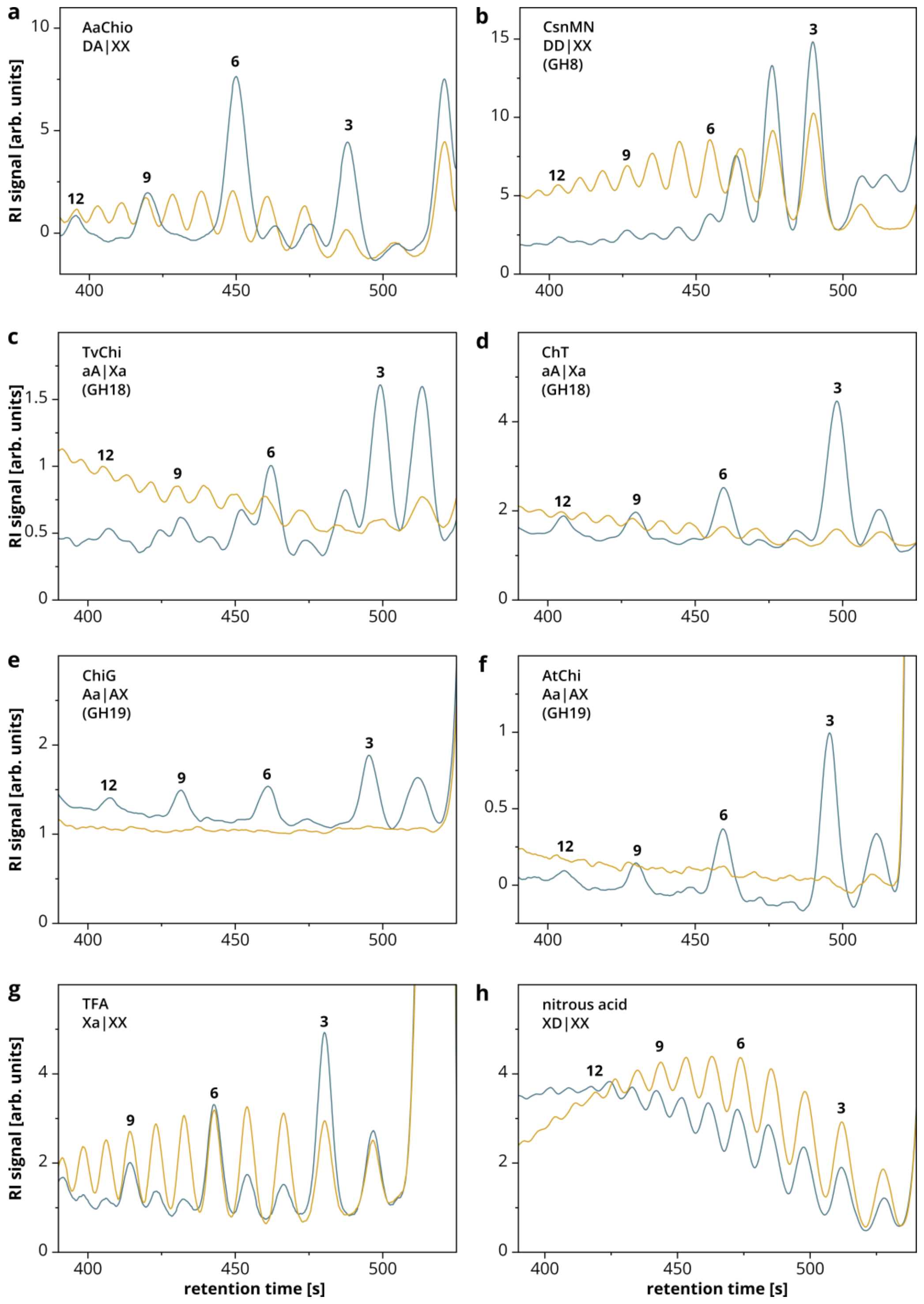

Supplementary Figure 7: Enzymatic or chemical cleavage products of HTDA (blue) and CNA (yellow) chitosans (FA = 0.17, M_w of ~110 kDa). Details of the chitosan samples are provided in Supplementary Table 1 (HTDA, 80/20; CNA, 134_0.19). The refractive index (RI) signals of oligomer products with the indicated DP are shown after size exclusion chromatography. The chitosans were enzymatically hydrolyzed using **a.** chitinosanase from *Alternaria alternata*[7] (AaChio), **b.** chitosanase from *Bacillus* sp. MN[8–10] (CsnMN), **c.** chitinase from *Trichoderma virens*[11] (TvChi), **d.** human chitotriosidase[12–14] (ChT), **e.** chitinase from *Streptomyces coelicolor* A3(2)[15] (ChiG) and **f.** chitinase from *Arabidopsis thaliana* (TAIR: AT3G54420) (AtChi), or partially chemically degraded by **g.** acidic hydrolysis using trifluoroacetic acid (TFA), or **h.** nitrous acid deamination. The subsite preferences of the enzymes from subsite -2 to +2 are indicated by X (no strong preference), A (absolute specificity for A), a (considerable preference for A), D (absolute specificity for D) or d (considerable preference for D). Preferential chemical acidic hydrolysis after A-units[16,17] by TFA (corresponding to a considerable A-preference at subsite -1) as well as the cleavage by nitrous acid deamination[18,19] exclusively after D-units[20] (corresponding to an absolute D-specificity at subsite -1) are indicated accordingly. HTDA, heterogeneously deacetylated; CNA, chemically *N*-acetylated.

Samples for elicitation assay in *Arabidopsis thaliana*

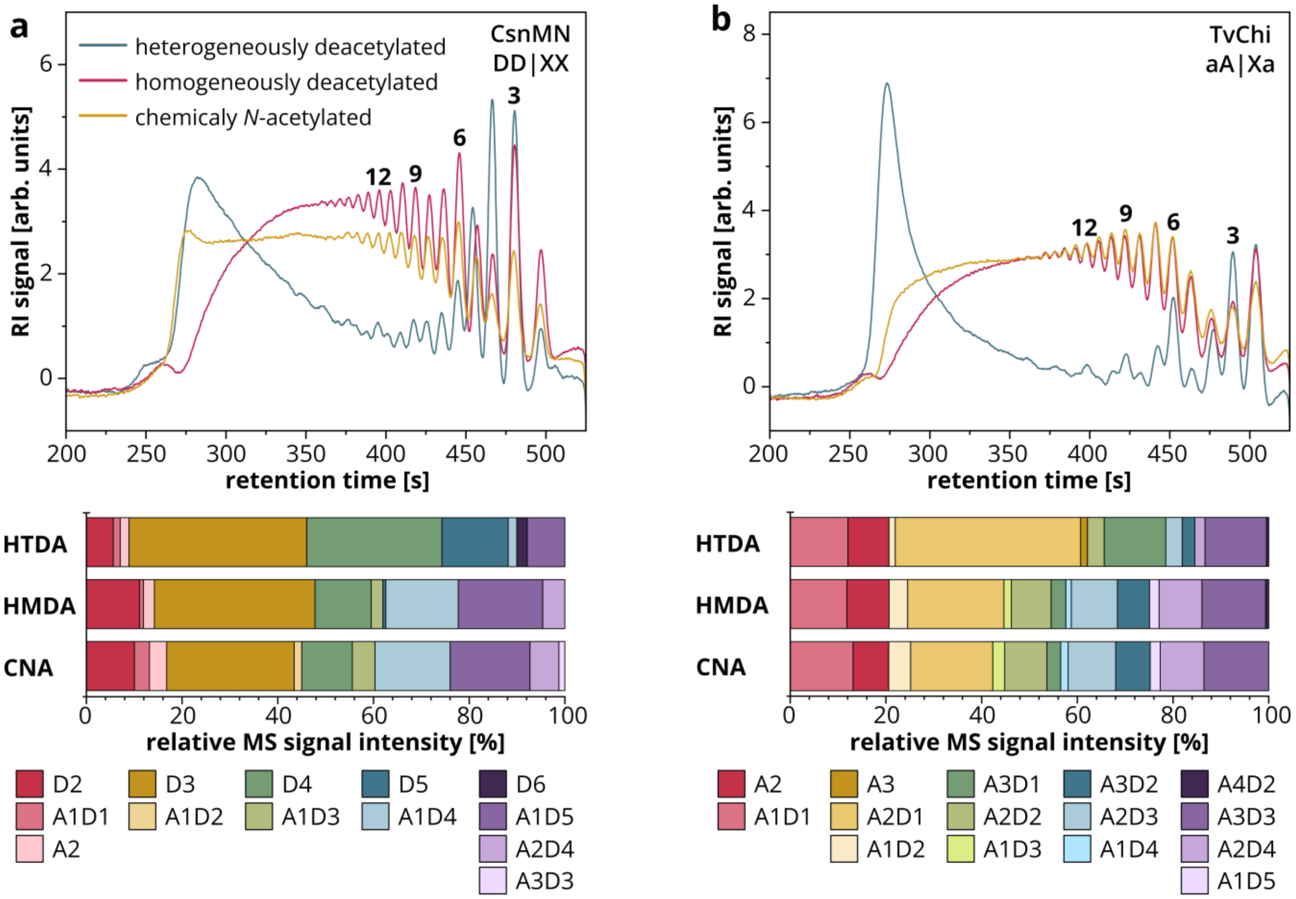

Supplementary Figure 8: Products of HTDA, HMDA and CNA chitosans (FA = 0.32) digested with different enzymes. Details of the chitosan samples are provided in Supplementary Table 1 (HTDA, shrimp_100min; HMDA, Viscosan_DDA69; CNA, 134_0.29). The refractive index (RI) signals of oligomer products with the indicated DP after size exclusion chromatography are shown on top, and profiles of the products with a DP of 2–6 are shown below. The signal intensity does not correlate with the actual amounts due to differences in ionization efficiency. **a.** Products of the chitosanase CsnMN[8–10]. **b.** Products of the chitinase TvChi[11]. The subsite preferences of the enzymes from subsites -2 to +2 are indicated with X (no strong preference), A (absolute specificity for A), a (considerable preference for A), D (absolute specificity for D) or d (considerable preference for D). HTDA, heterogeneously deacetylated; HMDA, homogeneously deacetylated; CNA, chemically *N*-acetylated.

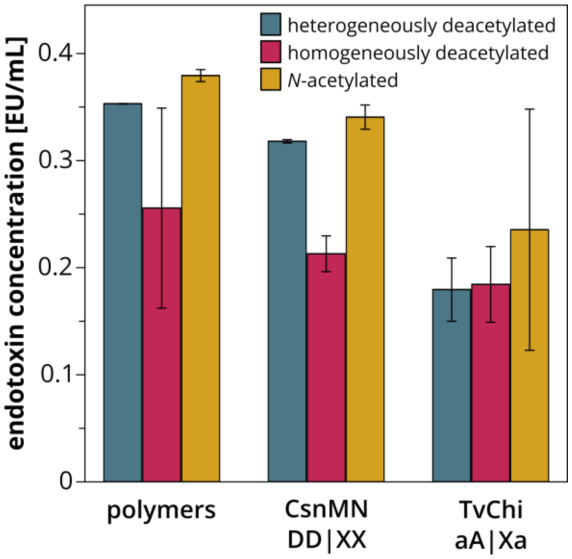

Supplementary Figure 9: Endotoxin concentrations in products of HTDA, HMDA and CNA chitosans (FA = 0.32) digested with different enzymes. Details of the chitosan polymers are provided in Supplementary Table 1 (HTDA, shrimp_100min; HMDA, Viscosan_DDA69; CNA, 134_0.29) which were digested with chitosanase CsnMN[8–10], or with chitinase TvChi[11]. The determined endotoxin concentration is given in endotoxin units (EU) per mL chitosan sample containing 100 µg/mL chitosan, which corresponds to the highest concentration tested in the elicitation assay in *Arabidopsis thaliana* (main article, Fig. 4). HTDA, heterogeneously deacetylated; HMDA, homogeneously deacetylated; CNA, chemically *N*-acetylated (N = 2).

Elicitation assay in potato (*Solanum tuberosum*) leaf discs

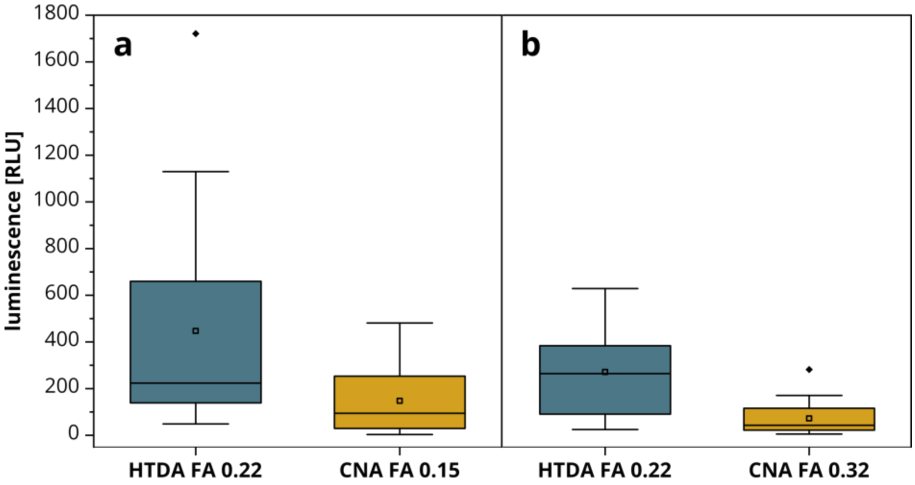

Supplementary Figure 10: Elicitation activity of differently produced chitosan polymers on potato (*Solanum tuberosum*) leaf discs. Details of the chitosan samples are provided in Supplementary Table 1 (HTDA FA 0.22, 651; CNA FA 0.15, 134_0.15_K; CNA FA 0.32, 134_0.32_K). Oxidative burst of **a.** CNA chitosan of FA 0.15, and **b.** CNA chitosan of FA 0.32, both shown in comparison to HTDA chitosan of FA 0.22 that was tested on the same 96-well plate each. All chitosans were tested at a concentration of 50 µg/mL. The oxidative burst was measured as a chemiluminescence signal resulting from the oxidation of luminol by reactive oxygen species. The box plots represent data of four independent experiments (N = 4) of four replicates each (n = 4), two outlier data points total were removed before based on the ROUT-method of GraphPad Prism with Q = 0.5%. The boxes span from the first to the third quartile, the median is shown as a horizontal line, the mean as a small square. The boundaries of the whiskers are based on the 1.5 interquartile range, values outside of these boundaries are plotted as outliers.

In silico modeling

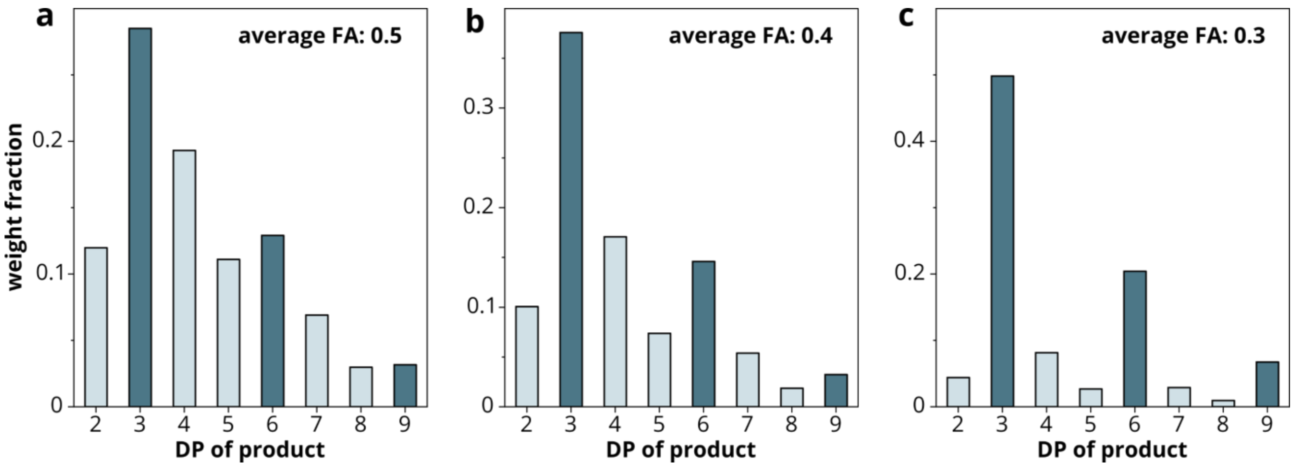

Supplementary Figure 11: Relative amounts of AaChio products with different DPs modeled *in silico*. The simulated full hydrolysis was performed with a cleavage specificity of DA|XX on 500 molecules each of DP 1000. The FA of all units at every third position (*pattern units*) is 0.75, but the average FA of the substrate is different: **a.** average FA = 0.5, **b.** average FA = 0.4, **c.** average FA = 0.3.

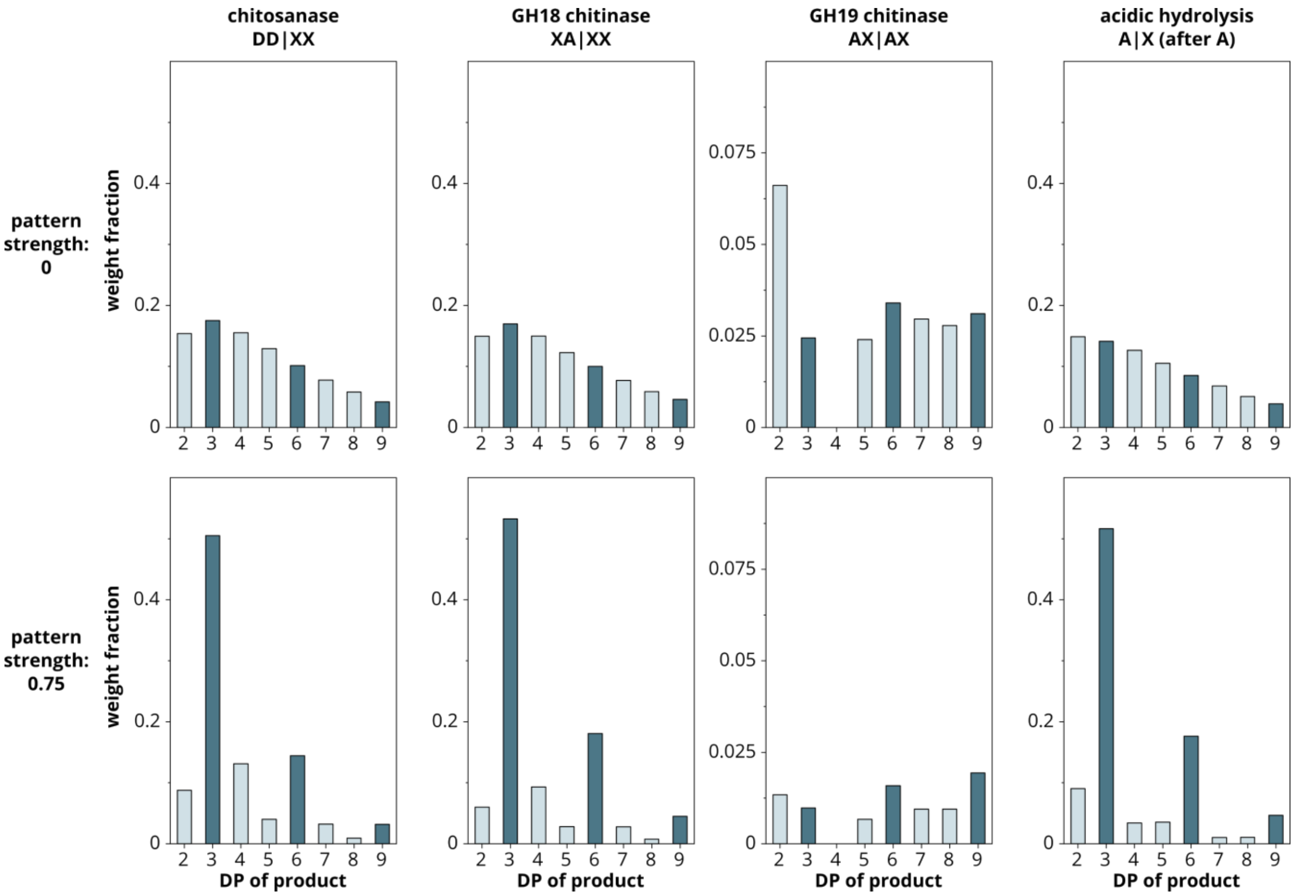

Supplementary Figure 12: Amounts of hydrolysis products with different DPs modeled *in silico*. The simulated hydrolysis was performed to completion on 500 molecules each of DP 1000. The average FA of each substrate is 0.32, but the strength of its $(DDA)_{DP/3}$ pattern is either 0 (top panel, random PA) or 0.75 (bottom panel). For each enzyme subsite from -2 to +2 either absolute specificities (A or D) or no specificity at all (X) were assumed, corresponding to the simplified subsite preferences of different enzyme families (columns 1–3), or to the acid hydrolysis preferentially occurring after A-units[16,17], here exclusively after A-units (column 4).

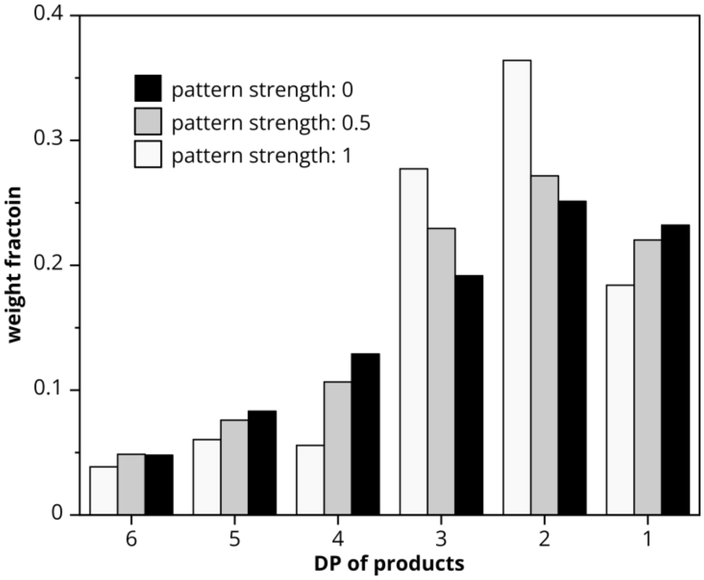

Supplementary Figure 13: Relative amounts of degradation products with different DPs following nitrous acid deamination modeled *in silico*. The simulated cleavage was performed after D-units to completion on 300 molecules each of DP 1000. The average FA of each substrate is 0.32, but the strength of its $(DDA)_{DP/3}$ pattern is 0 (random PA), 0.5 or 1. The layout and coloring of the figure are similar to those used in a publication on nitrous acid deamination[18].

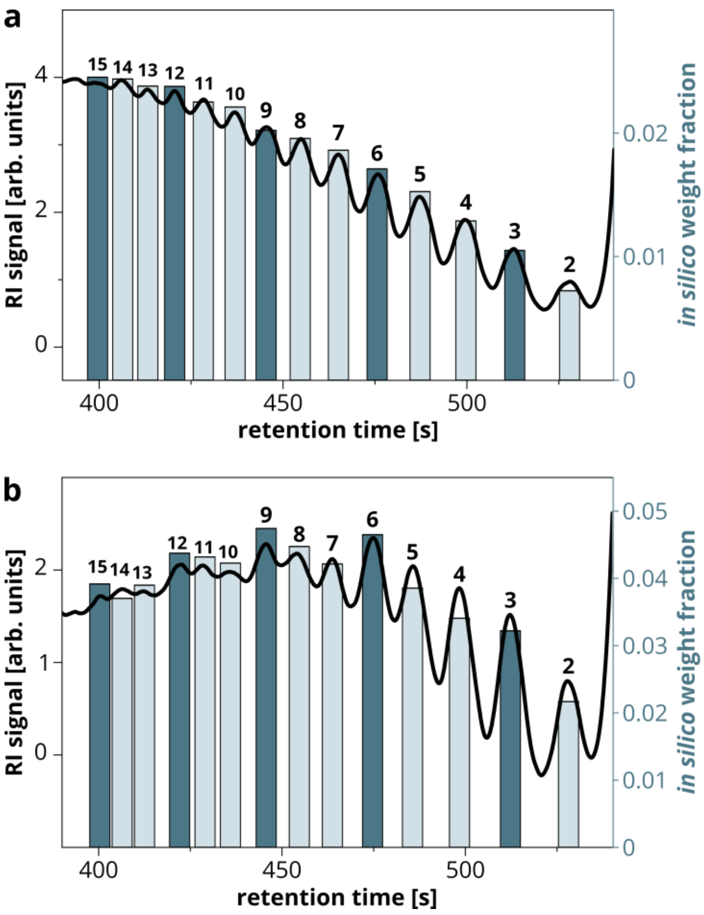

Supplementary Figure 14: Nitrous acid deamination products measured *in vitro* and modeled *in silico*. The black line plots exhibit the experimentally measured RI signals of the nitrous acid deamination products after size exclusion chromatography (numbers above peaks indicate the DP) of **a.** CNA substrate, or **b.** HTDA substrate; the same signals are plotted in Figure 3h. The bars show relative amounts of nitrous acid deamination products with different DP modeled *in silico*. A simulated cleavage was performed with a cleavage specificity of XD|XX on 1000 molecules each of DP 800 and with an average FA of 0.32. The strength of the $(DDA)_{DP/3}$ pattern and the number of cleavages were varied. **a.** The pattern strength of 0 corresponds to a completely random PA, and each molecule was cleaved 50 times. **b.** The pattern strength of 0.4 results in a moderate overrepresentation of A-units at every third position, and each molecule was cleaved 90 times.

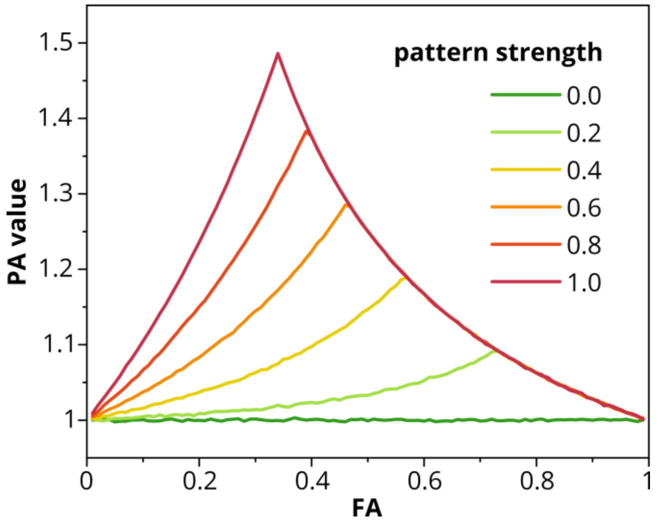

Supplementary Figure 15: PA values over the entire FA range for different pattern strengths modeled *in silico*. The pattern strength corresponds to the degree of overrepresentation of A-units at every third unit. For each pattern strength, the PA value was calculated for each FA between 0 and 1 with a step size of 0.01 by modeling 1000 molecules each of DP 1000 per data point. The equations used to calculate the PA value are described elsewhere.[21]

Supplementary Table 3: Comparison of experimental and *in silico* modeled triad frequencies. The first row shows *in vitro* data[22] for a HTDA chitosan sample (FA = 0.27). The other rows show *in silico* simulated frequencies of the different triads (F_{triad}) for chitosan populations of 1000 molecules each of DP 1000 with an average FA of 0.27 and a random to extreme $(DDA)_{DP/3}$ PA (pattern strengths 0–1).

	F_{AAA}	F_{AAD}	F_{DAA}	F_{ADA}	F_{ADD}	F_{DDA}	F_{DAD}	F_{DDD}
**Experimental**	0.02	0.07	0.06	0.11	0.13	0.13	0.12	0.36
**Pattern strength 0**	0.02	0.05	0.05	0.05	0.14	0.14	0.14	0.39
**Pattern strength 0.25**	0.02	0.05	0.05	0.05	0.15	0.15	0.15	0.38
**Pattern strength 0.5**	0.01	0.05	0.05	0.05	0.17	0.17	0.17	0.35
**Pattern strength 0.75**	0.00	0.03	0.03	0.03	0.20	0.20	0.20	0.29
**Pattern strength 1**	0.00	0.00	0.00	0.00	0.26	0.26	0.26	0.20

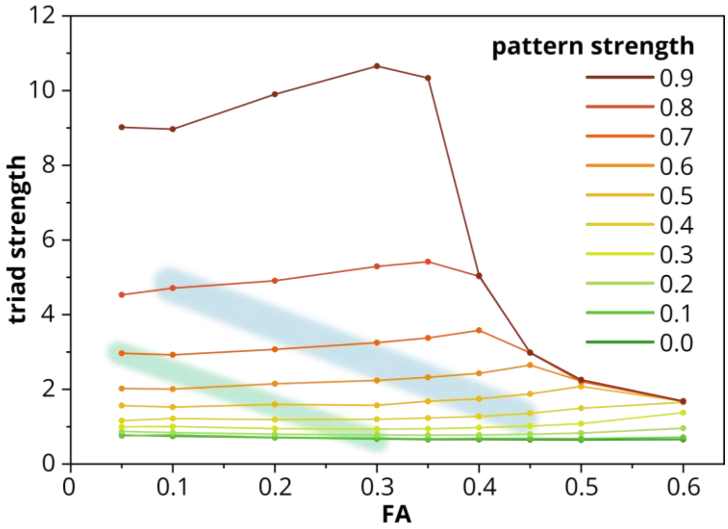

Supplementary Figure 16: Triad strength of *in silico* modeled AaChio products on chitosans of different FA and pattern strength. The pattern strength corresponds to the degree of overrepresentation of A-units at every third unit. For each pattern strength, the triad strength was calculated for each FA based on modeled AaChio (cleavage specificity: DA|XX) products on 1000 molecules each of DP 500 per data point. The blue and green highlighted areas correspond to the highlighted areas in Figure 2 for heterogeneously deacetylated shrimp chitin or squid chitin samples, respectively.

Conditions of enzymatic hydrolysis

Supplementary Table 4: Conditions for enzymatic hydrolysis of the chitosans produced by different methods (FA = 0.32 or 0.17). For each enzyme, the expression system, the tag used for purification by affinity chromatography, the enzyme concentration used for hydrolysis, and the buffer and incubation time used in the reaction are provided. TvChi: GH18 chitinase from *Trichoderma virens*[11]. ChT: GH18 human chitotriosidase[12–14]. ChiG: GH19 chitinase from *Streptomyces coelicolor* A3(2)[15]. AtChi: GH19 chitinase from *Arabidopsis thaliana* (TAIR: AT3G54420). CsnMN: GH8 chitosanase from *Bacillus* sp. MN[8–10]. NH_4Ac: ammonium acetate (Roth). TEA: triethanolamine (Sigma-Aldrich). HEK: human embryonic kidney (cells).

Enzyme	Expressed in	Purified by	Concentration [g/L]	Buffer	Incubation [days]
**TvChi**	*Pichia pastoris*	His_6 tag	0.010	NH_4Ac (200 mM), pH 4.2	5
**ChT**	HEK cells	Strep-tag II	0.020	NH_4Ac (200 mM), pH 4.2	1
**ChiG**	*Escherichia coli*	Strep-tag II	0.035	NH_4Ac (200 mM), pH 4.2	1
**AtChi**	*Nicotiana benthamiana*	Strep-tag II	0.895	TEA (65 mM), pH 8	FA 0.32: 6<br>FA 0.17: 4
**CsnMN**	*Escherichia coli*	Strep-tag II	0.010	NH_4Ac (200 mM), pH 4.2	3

References

1. Wattjes, J., Niehues, A. & Moerschbacher, B. M. Robust enzymatic-mass spectrometric fingerprinting analysis of the fraction of acetylation of chitosans. *Carbohydr. Polym.* **231**, 115684; doi:10.1016/j.carbpol.2019.115684 (2020).
2. Urs, M. J., Moerschbacher, B. M. & Cord-Landwehr, S. Quantitative enzymatic-mass spectrometric analysis of the chitinous polymers in fungal cell walls. *Carbohydr. Polym.* **301**, 120304; doi:10.1016/j.carbpol.2022.120304 (2023).
3. Schatz, C., Viton, C., Delair, T., Pichot, C. & Domard, A. Typical Physicochemical Behaviors of Chitosan in Aqueous Solution. *Biomacromolecules* **4**, 641–648; doi:10.1021/bm025724c (2003).
4. Sørbotten, A., Horn, S. J., Eijsink, V. G. H. & Vårum, K. M. Degradation of chitosans with chitinase B from Serratia marcescens. *FEBS J.* **272**, 538–549; doi:10.1111/j.1742-4658.2004.04495.x (2005).
5. Sannan, T., Kurita, K. & Iwakura, Y. Studies on Chitin, 2: Effect of Deacetylation on Solubility. *Die Makromol. Chemie* **177**, 3589–3600; doi:10.1271/nogeikagaku1924.23.437 (1976).
6. Wattjes, J. *et al.* Enzymatic Production and Enzymatic-Mass Spectrometric Fingerprinting Analysis of Chitosan Polymers with Different Nonrandom Patterns of Acetylation. *J. Am. Chem. Soc.* **141**, 3137–3145; doi:10.1021/jacs.8b12561 (2019).
7. Kohlhoff, M. *et al.* Chitinosanase: A fungal chitosan hydrolyzing enzyme with a new and unusually specific cleavage pattern. *Carbohydr. Polym.* **174**, 1121–1128; doi:10.1016/j.carbpol.2017.07.001 (2017).
8. Weikert, T., Niehues, A., Cord-Landwehr, S., Hellmann, M. J. & Moerschbacher, B. M. Reassessment of chitosanase substrate specificities and classification. *Nat. Commun.* **8**, 1698; doi:10.1038/s41467-017-01667-1 (2017).
9. Regel, E. K., Weikert, T., Niehues, A., Moerschbacher, B. M. & Singh, R. Protein-engineering of chitosanase from Bacillus sp. MN to alter its substrate specificity. *Biotechnol. Bioeng.* **115**, 863–873; doi:10.1002/bit.26533 (2018).
10. Gercke, D., Regel, E. K., Singh, R. & Moerschbacher, B. M. Rational protein design of Bacillus sp. MN chitosanase for altered substrate binding and production of specific chitosan oligomers. *J. Biol. Eng.* **13**, 23; doi:10.1186/s13036-019-0152-9 (2019).
11. Bußwinkel, F., Goñi, O., Cord-Landwehr, S., O'Connell, S. & Moerschbacher, B. M. Endochitinase 1 (Tv-ECH1) from Trichoderma virens has high subsite specificities for acetylated units when acting on chitosans. *Int. J. Biol. Macromol.* **114**, 453–461; doi:10.1016/j.ijbiomac.2018.03.070 (2018).
12. Eide, K. B. *et al.* Human Chitotriosidase-Catalyzed Hydrolysis of Chitosan. *Biochemistry* **51**, 487–495; doi:10.1021/bi2015585 (2012).
13. Eide, K. B., Lindbom, A. R., Eijsink, V. G. H., Norberg, A. L. & Sørlie, M. Analysis of productive binding modes in the human chitotriosidase. *FEBS Lett.* **587**, 3508–3513; doi:10.1016/j.febslet.2013.09.004 (2013).
14. Gorzelanny, C., Pöppelmann, B., Pappelbaum, K., Moerschbacher, B. M. & Schneider, S. W. Human macrophage activation triggered by chitotriosidase-mediated chitin and chitosan degradation. *Biomaterials* **31**, 8556–8563; doi:10.1016/j.biomaterials.2010.07.100 (2010).
15. Heggset, E. B., Hoell, I. A., Kristoffersen, M., Eijsink, V. G. H. & Vårum, K. M. Degradation of chitosans with chitinase G from streptomyces coelicolor A3(2): Production of chito-oligosaccharides and insight into subsite specificities. *Biomacromolecules* **10**, 892–899; doi:10.1021/bm801418p (2009).
16. Vårum, K. M., Ottøy, M. H. & Smidsrød, O. Acid hydrolysis of chitosans. *Carbohydr. Polym.* **46**, 89–98; doi:10.1016/S0144-8617(00)00288-5 (2001).
17. Einbu, A., Grasdalen, H. & Vårum, K. M. Kinetics of hydrolysis of chitin/chitosan oligomers in concentrated hydrochloric acid. *Carbohydr. Res.* **342**, 1055–1062; doi:10.1016/j.carres.2007.02.022 (2007).
18. Sashiwa, H., Saimoto, H., Shigemasa, Y. & Tokura, S. N-Acetyl group distribution in partially deacetylated chitins prepared under homogeneous conditions. *Carbohydr. Res.* **242**, 167–172; doi:10.1016/0008-6215(93)80031-9 (1993).
19. Sashiwa, H., Saimoto, H., Shigemasa, Y., Ogawa, R. & Tokura, S. Distribution of the acetamide group in partially deacetylated chitins. *Carbohydr. Polym.* **16**, 291–296; doi:10.1016/0144-8617(91)90114-R (1991).
20. Hussain, I., Singh, T. & Chittenden, C. Preparation of chitosan oligomers and characterization: their antifungal activities and decay resistance. *Holzforschung* **66**, 119–125; doi:10.1515/HF.2011.130 (2012).
21. Kumirska, J. *et al.* Determination of the pattern of acetylation of chitosan samples: Comparison of evaluation methods and some validation parameters. *Int. J. Biol. Macromol.* **45**, 56–60; doi:10.1016/j.ijbiomac.2009.04.002 (2009).
22. Vårum, K. M., Anthonsen, M. W., Grasdalen, H. & Smidsrød, O. 13C-N.m.r. studies of the acetylation sequences in partially N-deacetylated chitins (chitosans). *Carbohydr. Res.* **217**, 19–27; doi:10.1016/0008-6215(91)84113-S (1991).
